# The impact of tissue detection on diagnostic artificial intelligence algorithms in prostate digital pathology

**DOI:** 10.1038/s41598-026-52148-9

**Published:** 2026-05-13

**Authors:** Sol Erika Boman, Nita Mulliqi, Anders Blilie, Xiaoyi Ji, Kelvin Szolnoky, Einar Gudlaugsson, Emiel A.M. Janssen, Svein R. Kjosavik, José Asenjo, Marcello Gambacorta, Paolo Libretti, Marcin Braun, Radzislaw Kordek, Roman Łowicki, Kristina Hotakainen, Päivi Väre, Bodil Ginnerup Pedersen, Karina Dalsgaard Sørensen, Benedicte Parm Ulhøi, Lars Egevad, Kimmo Kartasalo

**Affiliations:** 1 Department of Medical Epidemiology and Biostatistics, Karolinska Institutet, Stockholm, Sweden; 2 Department of Pathology, Stavanger University Hospital, Stavanger, Norway; 3 Faculty of Health Sciences, University of Stavanger, Stavanger, Norway; 4 Department of Molecular Medicine and Surgery, Karolinska Institutet, Stockholm, Sweden; 5 The General Practice and Care Coordination Research Group, Stavanger University Hospital, Stavanger, Norway; 6 Department of Global Public Health and Primary Care, Faculty of Medicine, University of Bergen, Bergen, Norway; 7 Department of Pathology, Synlab, Madrid, Spain; 8 Department of Pathology, Synlab, Brescia, Italy; 9 Department of Pathology, Chair of Oncology, Medical University of Lodz, Lodz, Poland; 10 Department of Urology, Medical University of Lodz, Lodz, Poland; 11 Department of Clinical Chemistry and Hematology, University of Helsinki, Helsinki, Finland; 12 Laboratory Services, Mehiläinen Oy, Helsinki, Finland; 13 Department of Pathology, Mehiläinen Länsi-Pohja Hospital, Kemi, Finland; 14 Department of Radiology, Aarhus University Hospital, Aarhus, Denmark; 15 Department of Clinical Medicine, Aarhus University, Aarhus, Denmark; 16 Department of Molecular Medicine, Aarhus University Hospital, Aarhus, Denmark; 17 Department of Pathology, Aarhus University Hospital, Aarhus, Denmark; 18 Department of Chemistry, Bioscience and Environmental Engineering, University of Stavanger, Stavanger, Norway; 19 Institute for Biomedicine and Glycomics, Griffith University, Queensland, Australia; 20 Department of Oncology and Pathology, Karolinska Institutet, Stockholm, Sweden; 21 Department of Medical Epidemiology and Biostatistics, Karolinska Institutet, SciLifeLab, Stockholm, Sweden

**Keywords:** Prostate cancer, Cancer imaging, Computer science

## Abstract

Tissue detection is a crucial first step in most digital pathology applications. By applying image segmentation algorithms, all tissue is delineated and background discarded from further analyses, improving both computational efficiency and analytical results. Details of the segmentation algorithm are rarely reported, and there is a lack of studies investigating the downstream effects of a poor segmentation algorithm. Disregarding tissue detection quality could jeopardize patient safety if diagnostically relevant parts of the specimen are excluded from analysis in clinical applications. This study aims to determine whether performance of downstream tasks is sensitive to the tissue detection method, and to compare the performance of a classical and an AI-based tissue detection approach. To this end, we trained an AI model for Gleason grading of prostate cancer in whole slide images (WSIs) using two different tissue detection algorithms: thresholding (classical) and UNet++ (AI). A total of 33,823 WSIs scanned on seven digital pathology scanners were used to train the segmentation AI model. The downstream Gleason grading algorithm was trained and tested using 70,524 WSIs from 13 clinical sites scanned on 13 different scanners. On the slides where tissue could be detected by both algorithms, no significant difference in overall Gleason grading performance was observed. There was a decrease from 118 (0.43%) to 24 (0.09%) fully undetected tissue samples when switching from thresholding-based tissue detection to AI-based, suggesting this AI model may be more reliable than the classical model for avoiding total failures on slides with unusual appearance. Moreover, tissue detection dependent clinically significant variations in AI grading were observed in 3.5% of malignant slides, highlighting the role of tissue detection for optimal clinical performance of diagnostic AI.

**Supplementary Information:**

The online version contains supplementary material available at10.1038/s41598-026-52148-9.

## Introduction

Digital pathology involves analyzing histopathological whole slide images (WSIs) using computer methods^1^. Performance of artificial intelligence (AI) within this field has become increasingly accurate, and is now approaching expert pathologist level in a wide variety of tasks. Notably, digital pathology has seen recent successes in the fields of diagnostics^2–5^, such as cancer classification and grading; genomics^6–11^, such as detecting the presence of microsatellite instability or specific genetic mutations; and most recently, prognostics^12,13^, foregoing intermediate diagnostic steps and predicting outcomes directly. With these developments, clinical application of AI systems has become relevant^14,15^, resulting in an increased need for quality and safety of these systems^16,17^.

An early step in a large number of digital pathology tasks involves segmentation of the region of interest (ROI)^3,7–11,13,18–24^. By extracting and analysing only the ROI, computation time is heavily reduced while ensuring only the relevant parts of the image are analyzed. In some cases the ROI is a specific part of the sample, such as nuclei, glands, epithelia or lumina, but in the most general case it represents all tissue distinguished from image background. The general problem of segmentation involves categorizing each pixel of an image into different classes or objects. In this paper, “tissue detection” refers to binary tissue segmentation, where all tissue pixels constitute the ROI.

Segmentation tasks typically require manual labeling by a pathologist or the use of AI models, the latter of which requires training labels. The labels used for training such an AI model may not necessarily need to be of the same high quality that manual labeling provides, since such models are robust to label noise^25^. Tissue detection, however, is often simple enough to be done automatically using classical image analysis techniques such as thresholding or edge detection, as is common practice^3,7–10,13,19–21,24,26^. However, these methods also rely on user-defined parameters, the optimal choice of which may vary greatly depending on characteristics of the WSIs, such as the scanner used to digitize them. Indeed, generalisation across scanners, labs, and patient populations is crucial even at the tissue detection step. Despite being a frequent processing step, it is not common practice to include segmentation parameters in digital pathology articles, and some papers even omit the tissue detection method entirely^27,28^.

Several open source digital pathology pipelines that include tissue detection have been published^29–32^,sometimes arguing for the standardized use of these algorithms within the field of digital pathology, but these also fail to report tissue detection accuracy, and none of these algorithms have become standard^33^. Comparisons of tissue detection methods exist^34^, but we are not aware of investigations into their downstream performance effects in larger AI systems. Because a failure to detect tissue could, in the worst case, lead to exclusion of malignant tissue from analysis, consistent tissue detection is crucial in a clinical context.

Our main hypothesis was that as diagnostic AI models become increasingly precise, the overall performance of a diagnostic system risks being constrained by the quality of the initial tissue detection step. We tested this hypothesis in the context of a case study on Gleason grading of prostate cancer in biopsies, using a state-of-the-art AI model^35^. For the tissue detection step of the system, we compared a classical algorithm using Otsu’s thresholding^36^ and an AI segmentation method. The former has been developed in-house and used in its original and variant forms extensively over several years^3,10,37–41^, while the latter approach has been developed recently in view of planned clinical implementation and has been used in several validation studies^42–45^. We compared the two methods both in terms of directly measuring tissue detection performance and in terms of the resulting downstream performance of the Gleason grading AI which was developed in accordance with a pre-specified study protocol^46^.

## Methods

### Study design

The study had two distinct steps: development and evaluation of an AI tissue detection algorithm, and evaluation of downstream performance of a Gleason grading algorithm, comparing the AI tissue detection with that of a classical thresholding-based tissue detection method. The former was done by procuring an evaluation set of high quality tissue segmentation masks to compare against, then empirically finding a high performing architecture and training set (more details below). The latter utilized an end-to-end Gleason grading model presented recently^35^. A separate study protocol^46^ provides details on the development and evaluation of the Gleason grading model, including how reference grading by pathologists was obtained for each cohort.

Prior to conducting this study, we had generated segmentation masks for tissue detection for every WSI using Otsu’s thresholding, to be used for development of the Gleason grading algorithm^35^. The parameters of the thresholding algorithm had been selected individually for each cohort. These segmentation masks were used as labels for the training set of the AI tissue detection algorithm, which used a UNet + + architecture^47^. Subsets of the segmentation masks had been checked visually, and in certain cases manually edited to improve quality (see Table 1). Utilising some of these, as well as by iteratively re-running the thresholding algorithm with parameters tweaked on a WSI-by-WSI basis until high quality segmentation masks were confirmed by visual inspection, a set of 6,823 WSIs with high quality masks was generated. This set was used both for continuously validating the AI during training, as well as for evaluating its performance after finishing training for the purpose of model selection. For model selection, the model that achieved the best sensitivity was chosen, so long as the precision was not deemed unacceptable (< 90%).

**Table 1:** Summary of data and partitions for training, validation and evaluation for development of the segmentation model. The labels were considered strong if tissue segmentation masks from these cohorts had been checked visually to verify their correctness, and weak if the quality of almost all segmentation masks in the cohort had not been checked. Certain cohorts that had been checked visually had also had a small number of segmentation masks manually edited to improve the labels, the number of which is documented in the rightmost column.

Split	Cohort	Number of patients	Scanner	Number of WSIs	Label strength	Number of manually edited labels
Training	Stockholm3	2,444	Aperio	2,196	Strong	34 (1.5%)
			Hamamatsu	3,976	Strong	10 (0.25%)
			Philips	23,501	Weak	0 (0%)
	Stavanger University Hospital	639	Hamamatsu	4,150	Weak	0 (0%)
Validation	Stockholm3	116	Aperio	131	Strong	N/A
			Hamamatsu	874		
			Philips	1,146		
	Stavanger University Hospital	30	Hamamatsu	201		
	Radboud University Medical Center	33	3DHISTECH	142		
	Capio S: t Göran Hospital	5	Aperio	25		
			Hamamatsu	4		
Evaluation	Stockholm3	179	Aperio	225		
			Hamamatsu	1314		
			Philips	1661		
	Stavanger University Hospital	41	Hamamatsu	254		
	Radboud University Medical Center	137	3DHISTECH	516		
	Karolinska University Hospital	73	Hamamatsu	330		

Two sets of segmentation masks were generated for each WSI in the test set of the Gleason grading AI model. One set was generated using thresholding, using a single set of manually fine-tuned parameters for all WSIs. The other set was generated by running the segmentation AI model from the previous step, with no additional processing steps. The Gleason grading algorithm was then evaluated twice, once with tissue detection based on each set of segmentation masks. To allow for a direct comparison of downstream task performance, only those WSIs where both segmentation algorithms detected tissue were included. This excluded 140 difficult slides where one or both algorithms failed to detect any tissue, see Table 2 for details.

**Table 2:** The number of WSIs in the test cohorts of the downstream Gleason grading model where tissue segmentation by AI, by thresholding or by both methods failed to detect any tissue. Only WSIs with tissue detected by both algorithms were included in the comparison (Fig. 2).

Cohort	Tissue detection failure by AI only (*n* WSIs)	Tissue detection failure by thresholding only (*n* WSIs)	Tissue detection failure by both AI and thresholding (*n* WSIs)	Total slides
Aarhus University Hospital	0	0	0	102
Hospital Wiener Neustadt	0	0	0	50
Medical University of Lodz	0	79	8	2,435
Mehiläinen Länsi-Pohja*	0	0	0	1,963
Radboud University Medical Center	0	0	0	516
Spear Prostate Biopsy 2020**	0	8	0	2,570
Stavanger University Hospital	0	1	1	1,199
Stockholm3	0	6	1	14,907
Synlab Finland*	0	2	0	536
Synlab France	0	2	0	515
Synlab Switzerland*	2	18	12	2,429
University Hospital Cologne	0	0	0	50
**Total**	2 (< 0.01%)	116 (0.43%)	22 (0.08%)	27,272

*These cohorts were evaluated on location level.

**This cohort was evaluated on patient level.

### Datasets and data partitioning

#### Dataset for Gleason grading AI

A complete description of the development of the Gleason grading AI is given in the study protocol^46^, here we give a brief overview of the datasets involved. The dataset represents digitized hematoxylin and eosin (H&E) stained prostate core needle biopsies from patients who underwent biopsy between 2012 and 2023. Samples were obtained from 15 clinical sites, of which this study utilized slides from 13, excluding the Aquesta Uropathology (“AQ”) and Karolinska University Hospital morphological subtypes “KUH-2” cohorts representing non-gradable rare variants (see the study protocol for descriptions). The included slides were scanned using 13 whole slide scanners comprising 9 different models from 5 different vendors. The majority of these scanners use a 40x magnification, with a few using 20x magnification. The Gleason grading AI was trained on 55,798 WSIs (Stockholm3, “STHLM3”; Stavanger University Hospital, “SUH”) and tuned on 1,177 WSIs (STHLM3; Radboud University Medical Center, “RUMC”; Karolinska University Hospital, “KUH-1”). For this study, 27,272 WSIs were initially segmented. A small number of these were excluded due to missing information such as ISUP grading, and for cohorts with several WSIs per slide (STHLM3, SUH) only one WSI were chosen per slide, randomly. This resulted in an evaluation set for the Gleason grading AI of 18,848 WSIs (Aarhus University Hospital, “AUH”; Mehiläinen Länsi-Pohja, “MLP”; Medical University of Lodz, “MUL”; RUMC; Synlab Switzerland, “SCH”; Synlab Finland, “SFI”; Synlab France, “SFR”; Spear Prostate Biopsy 2020, “SPROB20”; STHLM3; SUH; University Hospital Cologne, “UKK”; Hospital Wiener Neustadt, “WNS”) from the internal and external validation cohorts. Internal validation cohorts represent data from the same lab and/or WSI scanner as the training data but from independent patients, and external validation cohorts represent data from different labs, scanners, and patients than the training data^46^. MLP, SCH and SFI were evaluated at a (anatomical) location aggregation level, and SPROB20 at a patient aggregation level, resulting in a final evaluation dataset of 13,549 unique cases.

#### Dataset for tissue detection AI

The WSIs for developing the segmentation AI model were a subset of the development set of the Gleason grading AI to ensure that the combined system respected the held-out internal and external test set splits specified in the study protocol. Multiple segmentation models were trained using a few different subsets of the data before choosing the UNet + + architecture trained on 33,823 WSIs (Table 1), as this achieved the highest tissue detection sensitivity on the segmentation evaluation set. Of these 33,823 WSIs, 6,172 (18.2%) had strong labels that were either checked visually to verify their quality, or, in 54 cases (0.16% of total), manually edited. The other 27,651 (81.8%) WSIs had weak labels that had not been checked for quality, apart from during the initial mask creation process when parameters were tuned empirically using a small and random subset of the WSIs. Validation for early stopping and evaluation for model selection used the set of 6,823 WSIs with manually curated high-quality segmentation masks (see “Study design”), split 30%−70% on patient-level (2,523 WSIs for validation and 4,305 WSIs for evaluation, see Table 1).

### Thresholding algorithm

The thresholding algorithm used for the comparison, to generate labels for training the segmentation AI model, and to generate the validation and evaluation sets, was based on Otsu’s method^36^ and subsequent morphological operations. For details on the specific functions used, see the supplementary materials. The original purpose of the implementation of the algorithm was to generate segmentation masks to be used for the Gleason grading AI, and these are the masks that constituted our labels. For these, parameters of the algorithm were chosen in a cohort-specific manner based on what was deemed optimal for each cohort. For the validation and evaluation sets of the tissue detection algorithm, the parameters were instead tuned to every individual WSI, and re-tuned until the visually evaluated quality of the mask was very high. Finally, for the comparison between thresholding and AI-based tissue detection, a single uniform set of parameters was used for all WSIs, selected manually based on what empirical testing revealed to be the most consistently good parameters during the validation set curation.

### Segmentation AI model

U-Net is a convolution neural network developed for segmentation of biomedical images^48^, and UNet + + is an extension of this architecture^47^. They are supervised learning models and hence require training data with annotated labels: WSIs or image patches with corresponding segmentation masks. The segmentation AI model architecture in this paper was that of UNet++, implemented using the SegmentationModels python library, version 0.3.3^49^. The implementation used encoder resnext101_32 × 4 d with a depth of 5, and five decoder channels (512, 256, 128, 54, 32). For details on augmentations, see the supplementary materials.

Training was done with an AdamW optimizer^50^ (torch.optim.AdamW) with a base learning rate of 1e-6, epsilon constant of 1e-6 for stability, and weight decay 0.01. Binary cross entropy (torch.nn.BCEWithLogitsLoss with pos_weight = 5.0) was used as a loss function for training, while F1-score (torchmetrics.classification.BinaryF1Score) was used as a metric on the validation set for early stopping.

### Tissue detection and patch extraction

For both the thresholding algorithm and segmentation AI, a resolution of 8.0 μm per pixel was used, which is heavily downsampled from the original resolution of the WSIs, and segmentation masks were stored as binary images. In training the segmentation AI, patches of size 512 × 512 pixels were extracted with no overlap. To fit an exact number of such patches, each WSI was first mirrored around each edge an appropriate amount. During inference, patches were generated such that they overlap 128 pixels about each edge to allow the edges of the predicted mask to be discarded. This avoids issues near tile edges due to lack of neighboring pixels providing context.

For both tasks, patches were downsampled from the closest higher resolution level in the WSI resolution pyramid using Lanczos resampling. For training the grading model, a higher resolution of 1.0 μm per pixel was used, with patches of size 256 × 256 pixels. Only patches with at least 10% of tissue pixels according to the segmentation masks were kept. Patches were extracted without overlap for training and with 128 pixel overlap during inference. Extracted patches were stored in TFRecord format, with each WSI saved as a separate file.

### Gleason grading model

The grading model used for evaluation in this study is a weakly-supervised algorithm relying on an attention-based multiple instance learning (ABMIL) architecture^51^. The model utilizes an EfficientNet-V2-S encoder^52^ initialized with ImageNet weights that produces patch-level feature embeddings. These are then aggregated into slide-level representations through the ABMIL and classified into primary and secondary Gleason patterns (i.e. 3, 4, or 5), and further translated into Gleason scores and International Society of Urological Pathology (ISUP) grades. The model was trained in an end-to-end fashion, jointly optimizing all model parameters for cross-entropy loss using the AdamW optimizer with a base learning rate of 0.0001. Details on the model design, hyperparameters, and complete training strategy are given in the original publication^35^. The model was trained on 10 cross-validation folds, stratified by patient and ISUP grade. During model predictions, test time augmentation (TTA) was applied on three iterations for each of the 10 folds, and the final predictions were obtained as a majority vote of the resulting 30 Gleason scores.

### Statistical analysis

The segmentation masks produced by thresholding and AI were compared using the pixel-wise metrics of sensitivity (true positive rate) and precision (positive predictive value). For our purposes, a model with high sensitivity is crucial, as low sensitivity indicates large missed regions. Precision is included to ensure that excessive amounts of background are not detected as tissue.

The Gleason grading model was trained using pre-existing segmentation masks generated with thresholding, and evaluated once using the UNet + + segmentation masks and once using masks generated with thresholding to detect tissue in the evaluation slides. In this step, all thresholding masks were created using a uniform set of thresholding parameters, generated via empirical testing. The Gleason grading models were compared using quadratic weighted Kappa, a modification of the Cohen’s Kappa statistic that measures agreement between two sets: in this case, the model’s predictions and the pathologists’ labels for each WSI or group of WSIs graded together. Confidence intervals were computed using bootstrapping with 1000 replicates.

## Results

### Tissue detection quality: thresholding vs. AI

For measuring tissue detection quality, we calculated pixel-level sensitivity and precision between the evaluation set masks and the AI and thresholding segmentation masks, respectively. The sensitivity was of highest importance, since a low sensitivity indicates that the tissue detection has mistakenly categorized tissue as background. Low precision instead indicates that large amounts of background have been categorized as tissue. Since the ground truth segmentation masks had been generated via a combination of thresholding and visual curation, in many cases with very similar or the same parameters as what was used in this comparison, thresholding-based masks were expected to have high mean accuracy. The primary purpose of this test was for model selection for the tissue detection AI and to ensure that the resulting model did not fall behind significantly on any metrics, especially sensitivity.

**Fig. 1:**
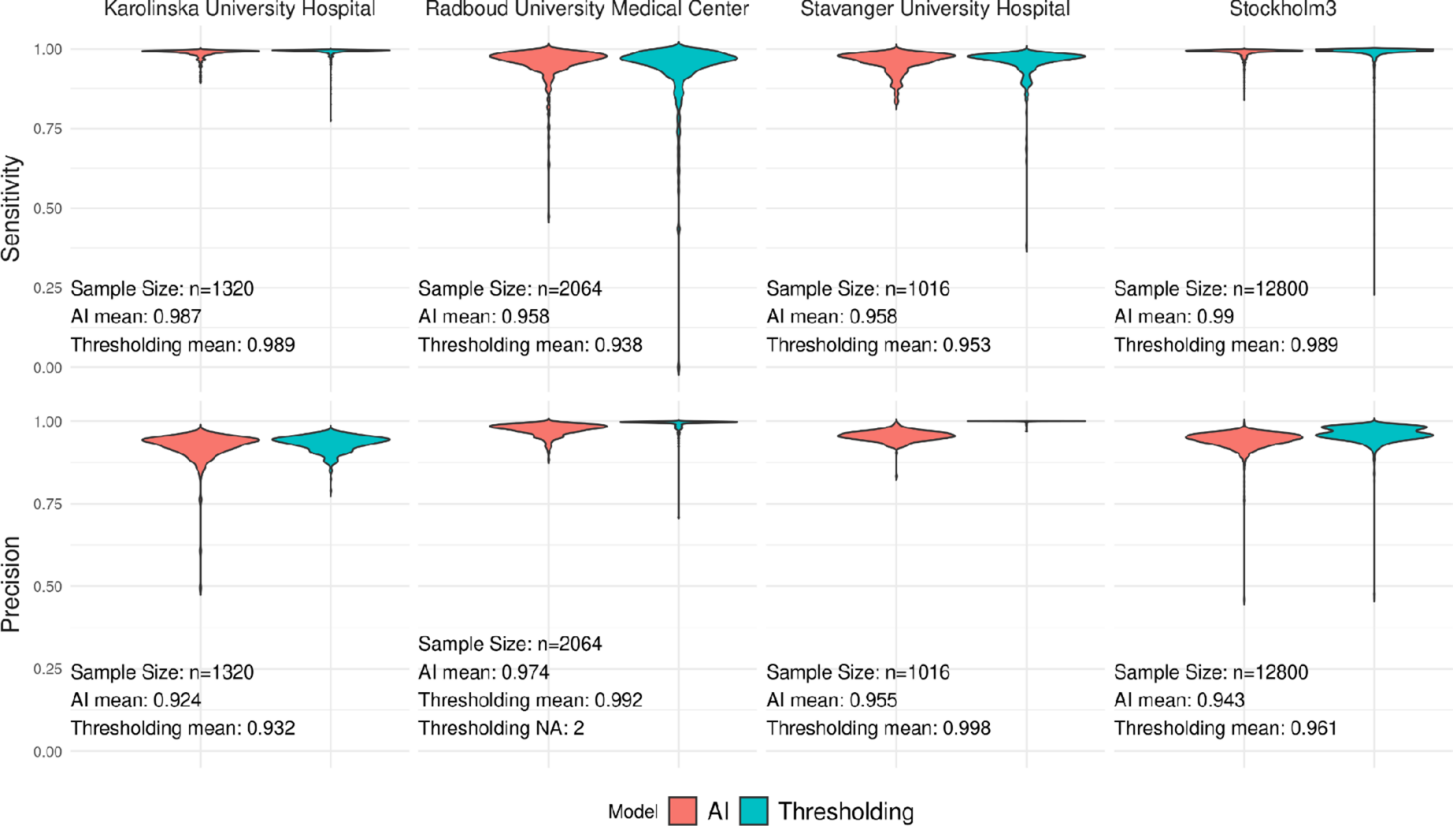
A violin plot of the pixel-wise sensitivity (top row) and precision (bottom row) of the tissue detection AI and the classical thresholding-based tissue detection. The ground truth tissue/background labels are curated high quality segmentation masks generated with thresholding.

The AI achieved an average sensitivity of 0.9840 (95% CI: 0.9833, 0.9848) and a precision of 0.9461 (95% CI: 0.9452, 0.9469) against the curated evaluation set masks, whereas the thresholding algorithm achieved 0.9804 (95% CI: 0.9790, 0.9819) and 0.9650 (95% CI: 0.9641, 0.9658), respectively. Two WSIs were excluded from calculating precision for the thresholding-based method due to not having any detected tissue. The distribution of sensitivity and precision for each cohort of the evaluation set can be seen in Fig. 1. Both algorithms performed highly but, importantly, the thresholding algorithm failed drastically in terms of sensitivity on a small set of individual WSIs (worst 5: 0.23, 0.38, 0.41, 0.42, 0.43), while the AI achieved more acceptable worst case performance (worst 5: 0.47, 0.63, 0.64, 0.69, 0.72).

The cohorts Karolinska University Hospital and Radboud University Medical Center were entirely unseen during training of the tissue detection AI. For the Karolinska University Hospital cohort, thresholding and AI tissue detection received almost the same mean sensitivity, 0.9889 (95% CI: 0.9865, 0.9913) and 0.9871 (95% CI: 0.9856, 0.9885), respectively. The thresholding model outperformed the AI model slightly on mean precision, 0.9317 (95% CI: 0.9290, 0.9344) and 0.9239 (95% CI: 0.9194, 0.9283) respectively. For Radboud University Medical Center, the AI achieved better mean sensitivity with 0.9576 (95% CI: 0.9535, 0.9617) versus 0.9379 (95% CI: 0.9297, 0.9462) for thresholding, but worse mean precision, 0.9745 (95% CI: 0.9730, 0.9760), versus 0.9921 (95% CI: 0.9907, 0.9935) for thresholding.

### Does tissue detection influence overall downstream grading performance?

The Gleason grading model trained using thresholding-based tissue detection masks was used to run predictions on the internal and external validation cohorts, using either thresholding- or AI-based tissue detection on these validation data. In the cases where one or both tissue detection models failed to detect tissue completely, the WSIs were discarded from the analysis. A total of 140 cases (0.5%) were discarded (Table 2). In summary, the failure rates and confidence intervals (calculated with exact binomial tests) were 24/27,272 (0.088%, 95% CI: 0.056%, 0.131%) for the segmentation AI model and 138/27,272 (0.506%, 95% CI: 0.425%, 0.598%) for the thresholding model, with a p-value from McNemar’s test for the difference on failure rates between the models less than 1e-10.

**Fig. 2:**
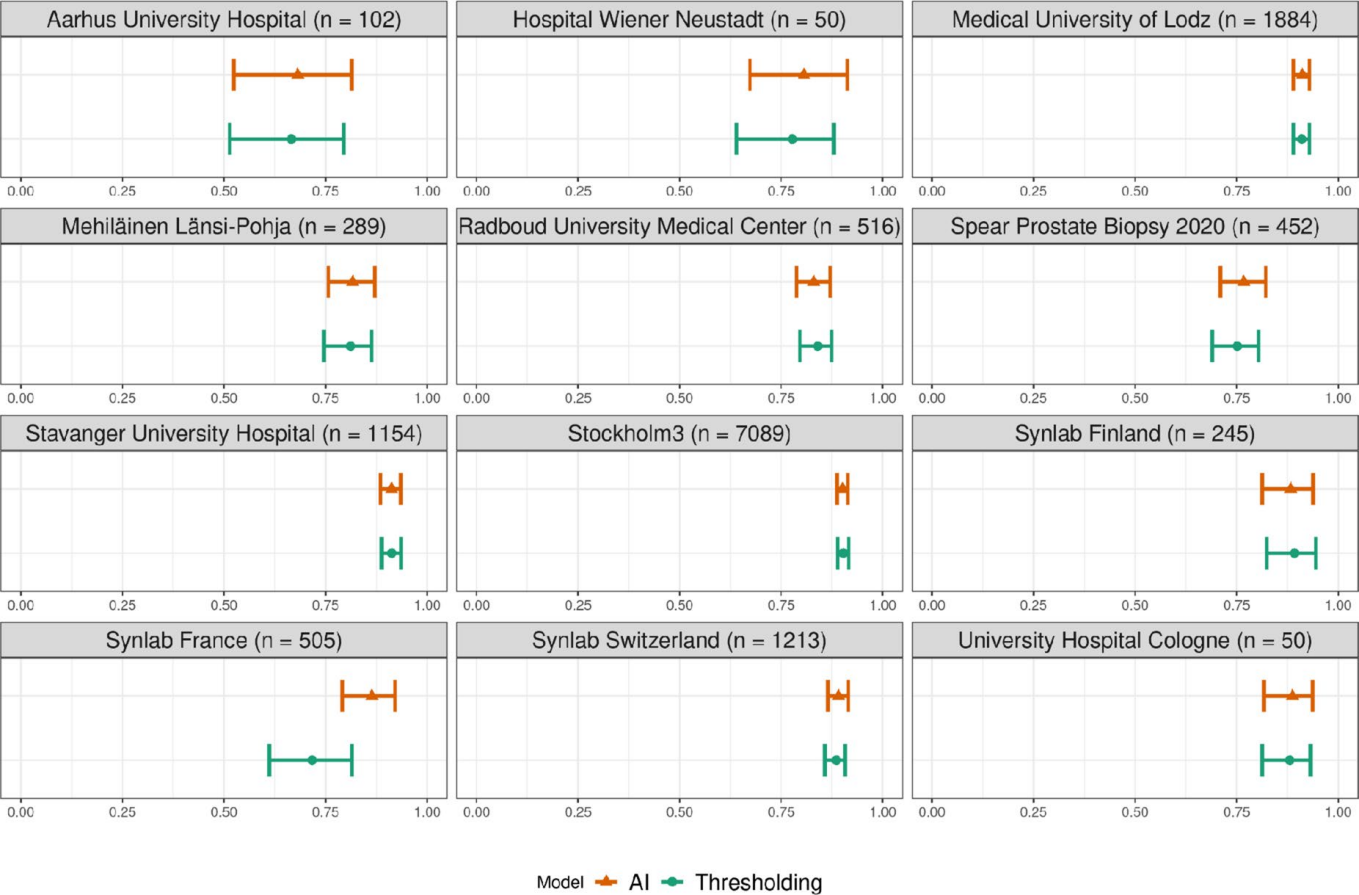
The concordance between the predictions of ISUP grade by the Gleason grading model and the reference grading by pathologists, measured by Cohen’s quadratically weighted kappa statistic. Results are shown based on tissue detection on the validation slides using AI-based and thresholding-based methods. The dots indicate point estimates on the entire dataset and the whiskers indicate 95% CIs. Confidence intervals were estimated with bootstrapping using 1000 replicates. Only cases where both models were able to detect any tissue were included, see Table 2. Synlab Finland, Synlab Switzerland and Mehiläinen Länsi-Pohja had reference grading per anatomical location and SPROB had reference grading per patient; WSIs from these cohorts were pooled to get predictions at location and patient levels, respectively.

For all WSIs where both tissue detection models identified any tissue, the quadratic kappa statistics quantifying the concordance between the ISUP grades predicted by the model and reported by the pathologists for each cohort are displayed in Fig. 2. No cohorts had non-overlapping confidence intervals, implying there is no large difference in overall performance between the two models. In Supplementary Fig. 1, a four-way comparison is shown which additionally includes a Gleason grading model trained on AI-based tissue detection masks.

In order to assess whether the relative significance of tissue detection on analyzed slides varies across models of varying performance, we repeated the experiment for all cohorts with slide-level reference grading available using an older grading model described in previous papers^3,37^. The model’s performance was heterogeneous across the cohorts, but for all cohorts the tissue detection model had insignificant impact on the total grading concordance. The result is shown in Supplementary Fig. 2.

### Tissue detection -dependent variations in AI Gleason grading

**Fig. 3:**
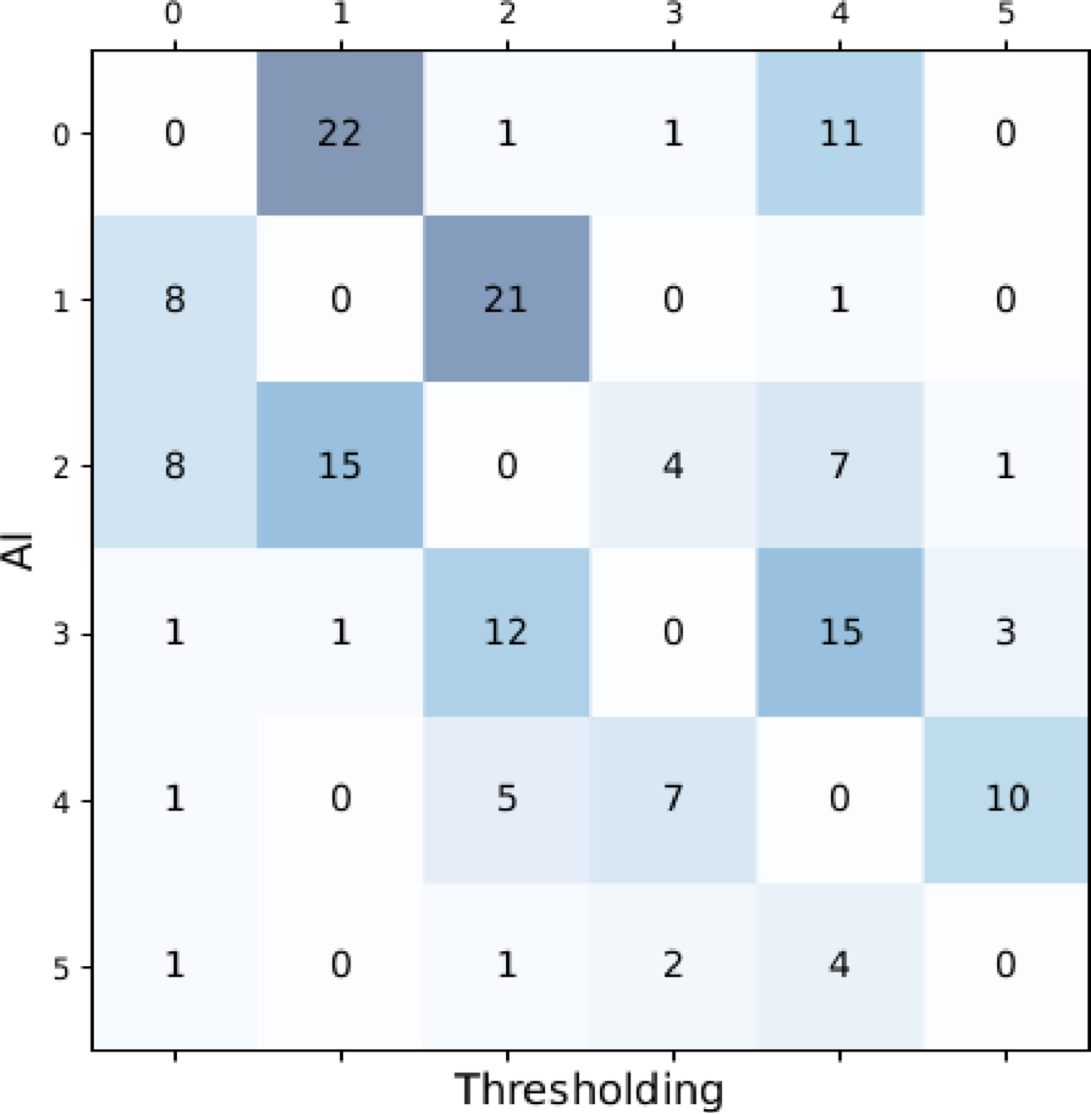
Confusion matrix showing the discordant ISUP grade predictions (with 0 indicating benign and 1–5 indicating ISUP grades) between the model evaluated using AI-based tissue detection masks versus thresholding-based tissue detection masks.

We identified all the slides with per-slide pathologist grading available, where the two tissue detection algorithms led to different predictions of ISUP grade during evaluation, using the same downstream grading model (trained on thresholding-based masks). This occurred for 163 slides out of 11,350 (1.4%), or 120 out of 3,459 (3.5%) malignant slides. In 44 of these 120 cases (36.7%), evaluating with the thresholding-based masks led to ISUP prediction matching the ground truth label but evaluating with the AI-based masks did not, and in 45 of the 120 cases (37.5%) the opposite was true (in the remaining cases, neither method led to predictions matching the ground truth labels). In Fig. 3, we show in a confusion matrix how the prediction differed between the two segmentation methods for all 163 discordant cases, regardless of their label.

**Fig. 4:**
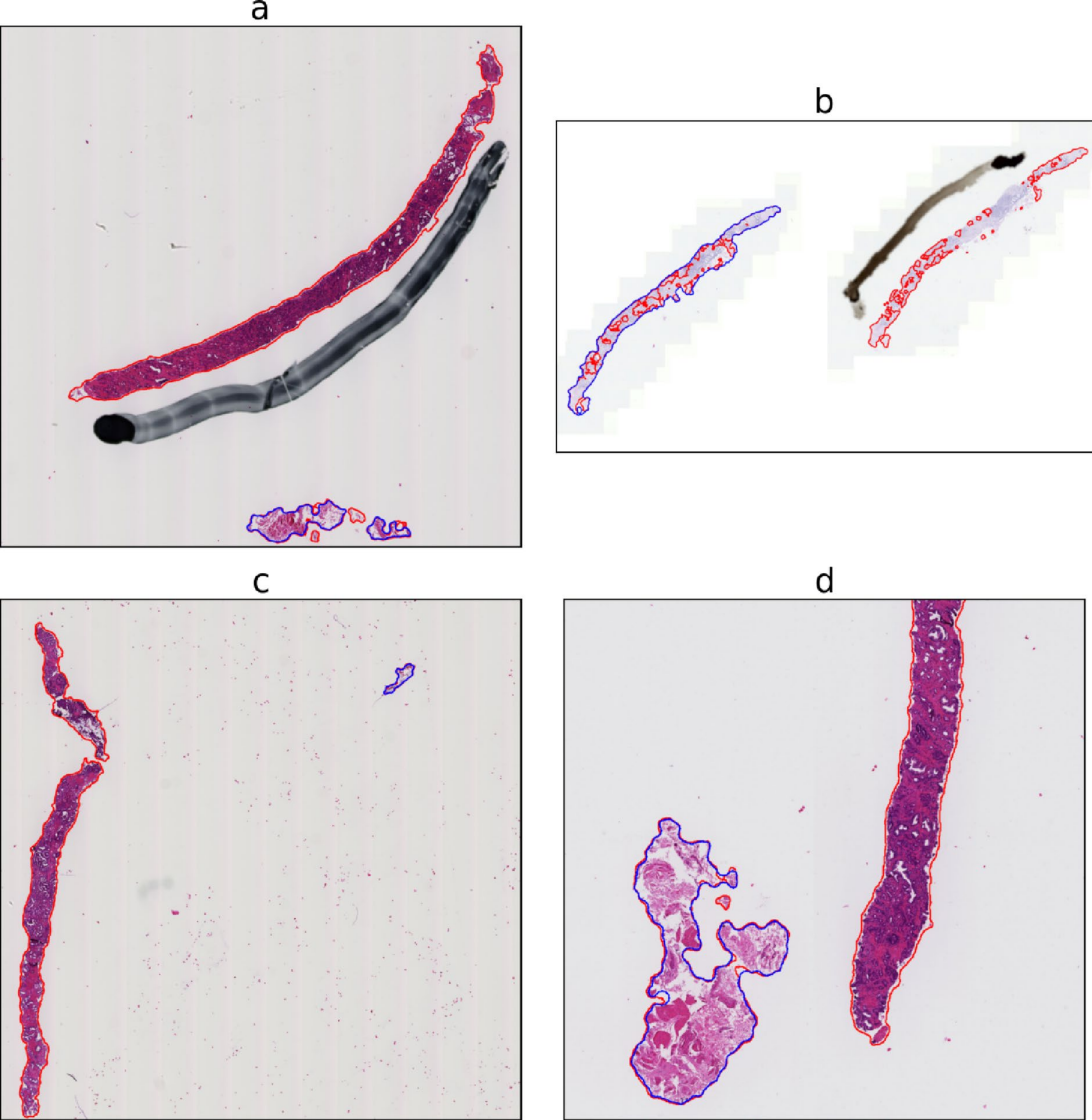
Outlines of tissue segmentation masks for AI (red) and thresholding (blue) in example cases where cancer grading by the downstream model was affected by differences in tissue detection. **(a)** The thresholding model missed an entire large piece of tissue and subsequently the Gleason grading model predicted ISUP 0 when it is in fact ISUP 2. **(b)** Both methods struggled to properly segment tissue on this slide: the thresholding model missed an entire piece of tissue while the segmentation AI model only detected some chunks. The Gleason grading AI is still able to predict ISUP 3, the correct grade, using the thresholding segmentation mask but predicts ISUP 2 using the AI segmentation mask. **(c)** Both models incorrectly segment some debris, but only the segmentation AI model detects the large piece of tissue. Subsequently, the Gleason grading algorithm predicts ISUP 0 using the thresholding mask and ISUP 3, the correct grade, using the AI mask. **(d)** Zoomed in for clarity. Both models segment a piece of tissue at the bottom but the thresholding segmentation completely fails to identify a much larger piece of tissue extending high above the edge of the cropped image. Subsequently, the Gleason grading AI predicts ISUP 0 when using the thresholding-based segmentation mask and ISUP 2, the correct grade, when using the AI mask.

Four example WSIs with the true label ISUP 2 or higher were chosen for visualization in Fig. 4. In each case, it is clear why the choice of tissue detection mask was important for making the correct prediction. This elucidates what may go wrong when using automated tissue detection. In a clinical setting, unless the system flags for potentially faulty tissue detection (which may be algorithmically challenging) or the tissue detection can be easily visualized by a pathologist, none of these cases would likely be detected as erroneous.

## Discussion

Many digital pathology projects utilize tissue detection as a computationally cheap way to remove background from analyses. This both saves compute time and physical energy resources, as well as reduces background-related problems such as shortcut learning^53^ due to pathologists’ pen markings. For these reasons, tissue detection is likely to remain relevant even for slide-level analytics and the digital pathology community should ensure the process is reliable, reproducible, and consistent.

The sensitivity distribution for the tissue detection models in Fig. 1 indicates that they both aptly caught tissue in the vast majority of cases, but the thresholding model missed large areas of tissue more often than the segmentation AI model. Since the cases where large pieces of tissue are missing are the most likely to result in failed cancer detection, improving the sensitivity of tissue detection was the most important task. Due to the partial circularity in generation of the ground truth masks using thresholding for this comparison, there was no expectation for the AI model to outperform the thresholding model in the vast majority of cases. What these results show is that the AI model does not fall behind significantly in mean performance, and that the worst-case performance of the evaluated AI model improves on that of the thresholding-based algorithm when the parameters are not ideally tuned.

The results in Fig. 2 indicate that no statistically significant overall difference in ISUP grade prediction performance relative to pathologist-provided reference grading could be observed between the two tissue detection approaches. Hence, our initial hypothesis that tissue detection can constitute a bottleneck for downstream systems was not confirmed. Conclusively testing this hypothesis may not be possible without even more powerful models, and thus remains a future prospect as models continue improving. The existence of outlier cases where the tissue detection was highly important is evident from Table 2; Fig. 4. In Table 3, we show summary statistics (sensitivity, specificity, AUC) for cancer detection (benign versus malignant), as well as quadratic weighted kappa for Gleason score prediction and ISUP prediction. Overall, tissue detection appears to not have enough impact to significantly improve mean accuracy over large datasets, but remains important for worst-case performance. While in certain cases, such as the cases shown in Fig. 4, it is clear why one model should fail and the other succeed, there are also cases where the masks are very similar but downstream prediction is different. Since the purpose of the tissue detection mask is to decide which tiles are used as input to the grading model (at least 10% of the pixels in a tile should be tissue in our implementation), the two masks in these cases lead to certain tiles near the edges of tissue either being included or not, influencing the downstream result. This indicates that models may, in certain edge cases, be sensitive to changes in input data, further corroborating that consistent and accurate tissue detection models are important and that this step should be considered an integral part of the overall analysis pipeline.

**Table 3:** Accuracy metrics for the Gleason grading AI when evaluated using AI-based or thresholding-based tissue detection masks, per cohort. Sensitivity, specificity and AUC refer to cancer prediction (malignant vs. not malignant). GS QWK is gleason score quadratic weighted kappa, and ISUP QWK is ISUP quadratic weighted kappa, the same which is shown graphically in Fig. 2.

Cohort	Number of samples	AI					Thresholding				
		Sensitivity (95% CI)	Specificity (95% CI)	AUC (95% CI)	GS QWK (95% CI)	ISUP QWK (95% CI)	Sensitivity (95% CI)	Specificity (95% CI)	AUC (95% CI)	GS QWK (95% CI)	ISUP QWK (95% CI)
Aarhus University Hospital	102	0.966 (0.915–1.000.915.000)	0.907 (0.816–0.978)	0.959 (0.914–0.992)	0.549 (0.324–0.783)	0.681 (0.524–0.814)	0.966 (0.914–1.000.914.000)	0.907 (0.805–0.980)	0.960 (0.916–0.991)	0.534 (0.319–0.774)	0.666 (0.514–0.795)
Hospital Wiener Neustadt*	50	0.960 (0.863–0.995)	N/A	N/A	N/A	0.806 (0.673–0.913)	0.960 (0.863–0.995)	N/A	N/A	N/A	0.778 (0.640–0.880)
Medical University of Lodz	1884	0.942 (0.921–0.962)	0.952 (0.940–0.963)	0.981 (0.972–0.989)	0.900 (0.876–0.921)	0.912 (0.890–0.929)	0.940 (0.919–0.961)	0.951 (0.939–0.961)	0.979 (0.969–0.987)	0.899 (0.876–0.921)	0.910 (0.890–0.929)
Mehiläinen Länsi-Pohja	289	0.936 (0.903–0.967)	0.957 (0.901–1.000.901.000)	0.971 (0.946–0.988)	0.799 (0.731–0.858)	0.817 (0.757–0.871)	0.936 (0.902–0.967)	0.957 (0.905–1.000.905.000)	0.971 (0.948–0.989)	0.790 (0.712–0.845)	0.812 (0.746–0.863)
Radboud University Medical Center	516	0.966 (0.947–0.984)	0.954 (0.921–0.983)	0.986 (0.974–0.995)	0.794 (0.743–0.840)	0.830 (0.788–0.871)	0.966 (0.943–0.984)	0.959 (0.931–0.984)	0.985 (0.974–0.995)	0.807 (0.755–0.848)	0.840 (0.796–0.874)
Spear Prostate Biopsy 2020**	452	0.931 (0.900–0.960)	0.920 (0.877–0.962)	0.808 (0.769–0.848)	N/A	0.767 (0.710–0.822)	0.938 (0.908–0.963)	0.914 (0.865–0.955)	0.746 (0.700–0.788.700.788)	N/A	0.751 (0.690–0.804)
Stavanger University Hospital	1154	0.936 (0.913–0.958)	0.970 (0.957–0.981)	0.992 (0.987–0.996)	0.876 (0.835–0.907)	0.913 (0.885–0.935)	0.938 (0.914–0.960)	0.970 (0.957–0.981)	0.992 (0.986–0.996)	0.876 (0.837–0.911)	0.913 (0.888–0.936)
Stockholm3	7089	0.940 (0.930–0.951)	0.973 (0.968–0.977)	0.988 (0.984–0.991)	0.879 (0.861–0.895)	0.902 (0.887–0.914)	0.939 (0.928–0.949)	0.972 (0.967–0.977)	0.987 (0.983–0.990)	0.882 (0.865–0.898)	0.903 (0.889–0.916)
Synlab Finland	245	0.899 (0.837–0.953)	0.973 (0.942–0.993)	0.953 (0.916–0.985)	0.895 (0.812–0.949)	0.883 (0.813–0.938)	0.919 (0.862–0.970)	0.980 (0.952–1.000.952.000)	0.956 (0.925–0.989)	0.900 (0.827–0.951)	0.892 (0.824–0.945)
Synlab France	505	0.970 (0.938–0.993)	0.943 (0.917–0.966)	0.993 (0.988–0.997)	0.857 (0.753–0.923)	0.864 (0.791–0.921)	0.978 (0.950–1.000.950.000)	0.916 (0.886–0.943)	0.988 (0.980–0.994)	0.716 (0.589–0.814)	0.717 (0.611–0.815)
Synlab Switzerland	1213	0.916 (0.887–0.943)	0.983 (0.973–0.991)	0.985 (0.974–0.992)	0.853 (0.915 − 0.887)	0.891 (0.865–0.915)	0.916 (0.886–0.942)	0.983 (0.973–0.990)	0.985 (0.975–0.993)	0.841 (0.798–0.880)	0.886 (0.858–0.907)
University Hospital Cologne*	50	0.960 (0.863–0.995)	N/A	N/A	N/A	0.887 (0.817–0.937)	0.960 (0.863–0.995)	N/A	N/A	N/A	0.881 (0.813–0.932)

*These cohorts have no benign samples in our data.

**This cohort did not have pathologist grading at Gleason level.

In both the comparison of segmentation performance as well as the comparison of downstream performance among samples where both segmentation approaches detected tissue, no clear advantage is evident for either tissue detection method. In terms of complete tissue detection failures, we observed a lower failure rate for the AI-based tissue detection model, especially for samples that stray from the typical appearance of these images. While total detection failures are relatively rare, decreasing them further may be advantageous, particularly in a clinically deployed system where even a low fraction of samples requiring intervention (e.g. manual delineation of tissue) by the pathologist end user may be experienced as cumbersome and adds an element of subjectivity and inter-observer variation to the process. In general, well-trained deep learning models have often been more reliable than classical methods across a variety of image analysis tasks, which is one of the reasons for their rise in popularity in the 2010s. However, the current study only compared one classical algorithm with one deep learning model, and while the choice of these two specific methods was well motivated by their established roles in the research group and prior studies, the results should not be taken as broadly representative of these two families of methods.

A limitation of the study is the lack of a standardized procedure for obtaining training data for the AI segmentation model. Naturally, we wanted to train on the highest quality segmentation masks we had available, which were not generated in a reproducible manner. We believe, however, that a high-performing U-Net type model can be trained without any complicated procedures for generating training labels, for instance by training it directly on the labels generated via a thresholding algorithm without manual refinement. In our own data, only 0.16% of masks had been manually refined, and the cohort-specific thresholding parameters did not considerably outperform the uniform parameters we used in this paper. This indicates that weak labels can be sufficient for training an AI segmentation model at least for relatively simple tasks like tissue detection. Furthermore, research groups utilising tissue segmentation will already have segmentation masks available to them, and those masks can constitute their training labels.

A second limitation is the partial circularity in the evaluation dataset used for the segmentation model. Since this dataset was produced using thresholding, the pixel-level comparisons between AI and thresholding tissue detection methods (Fig. 1) will favor thresholding, and in certain cases the ground truth mask and the thresholding-based mask in the comparison will be identical. This makes it very hard to compare the accuracy in the tissue detection masks directly, and is a large reason why it was important to also include downstream results.

A third limitation is that inclusion of samples in the analysis of downstream grading performance was conditional on tissue being detected by both tissue detection models. As no ISUP grade could be assigned by the downstream model in the absence of any input tissue, these samples could not be included in the calculation of grading performance metrics. In a clinically deployed system, such empty inputs could be flagged as errors, potentially recommended for rescanning. For the more dangerous case where large pieces of tissue are missed, the model may still produce an output. Hence, a visualization of the tissue detection for the pathologist to confirm that the input to the model appropriately captured all relevant tissue would be useful in clinical applications.

A fourth limitation is that we have not conducted a full cost-benefit analysis comparing the classical and AI-based tissue detection methods. Such an analysis would include in consideration not only the direct and indirect effects of different total failure rates and diagnostic discordances on pathologist end users and patients, but also factor in the development, maintenance and computational costs associated with different approaches.

A strength of this study is the large amount of data available. However, it is noteworthy that tissue detection can be learned with smaller models utilising less data as well, and we do not contend that training data amounts similar to the ones used in this study are necessary to incorporate AI tissue detection into a digital pathology pipeline. Since it is in the interest of the community to make models smaller and more efficient both to improve accessibility to clinics and to reduce carbon emissions^54^, future studies can examine training smaller tissue detection models. Another potential improvement of our model could be to train using more color augmentations to avoid issues where the AI overlearns color associations, which would make it more robust to unusual color profiles, such as the pale white tissue of Fig. 4b. This would also address potential overfitting issues, which could lead to an AI tissue detection model having complete failures for WSIs with a different profile from those seen during training.

In this paper, we have analyzed how the choice of tissue detection method can influence the computational pathology pipeline in a diagnostic prostate pathology system. Firstly, we did not observe significant tissue detection dependent effects on the overall downstream diagnostic or grading performance. We hypothesize that this question may need to be revisited in the future as downstream AI models become increasingly precise, which may increase the relative contribution of pre-processing steps like segmentation on total diagnostic error rates. Secondly, there was a significant difference in how often the diagnostic AI process malfunctions by failing to detect any tissue on a slide. In this respect, the evaluated AI tissue detection method was more reliable than a classical thresholding algorithm. Thirdly, differences in predicted cancer grading caused by different tissue detection methods were observed in 3.5% of malignant cases. As clinical application of these systems has become reality, it is increasingly important that every part of these algorithms works accurately and consistently to ensure the efficiency and patient safety of diagnostic AI in all situations. While there is not sufficient evidence to claim that either of the compared methods would be categorically superior over the other, this paper shows that this step of a computational pathology deep learning system deserves further attention.

## Supplementary Information

Below is the link to the electronic supplementary material.

Supplementary Material 1

Supplementary Material 2

Supplementary Material 3

Supplementary Material 4

## Data Availability

A subset of the data used for development (STHLM3 and RUMC cohorts) is available for non-commercial purposes subject to a CC BY-SA-NC 4.0 license as part of the PANDA challenge dataset and is freely downloadable after registration at https://www.kaggle.com/c/prostate-cancer-grade-assessment. This study also utilizes publicly available datasets used for external validation (SPROB20, UKK, and WNS). The SPROB20 cohort is available for non-commercial purposes under the AIDA BY license upon accepted access request at the AIDA Data Hub at *https://datahub.aida.scilifelab.se/*10.23698/aida/sprob20. UKK and WNS cohorts are available for non-commercial purposes under the CC BY-NC-SA 4.0 license upon accepted access request at https://zenodo.org/records/8102833 and https://zenodo.org/records/8102929. Data from additional sources cannot be shared publicly. For any requests to access these sources, inquiries should be directed to K.K. at Karolinska Institutet. Requests will be evaluated on a case-by-case basis, with approvals granted if they comply with data privacy regulations and intellectual property policies.

## Code availability

Core components of the segmentation and grading models relied on open-source repositories. For the grading model we used https://github.com/AMLab-Amsterdam/AttentionDeepMIL and https://github.com/huggingface/pytorch-image-models, and for the segmentation model we used https://github.com/qubvel-org/segmentation_models.pytorch. Torch (https://github.com/pytorch/pytorch*)* was used for model training and prediction. Scipy (https://github.com/scipy/scipy*)* and scikit-image (https://github.com/scikit-image/scikit-image*)* were used for the thresholding algorithm. Albumentations (https://github.com/albumentations-team/albumentations*)* was used for applying augmentations during model training. All steps involved in model training and design have been thoroughly documented in the Methods section to allow independent replication.
