# Supplementary material for: The impact of tissue detection on diagnostic artificial intelligence algorithms in prostate digital pathology
Source: Sci Rep. 2026 May 13;16:14968. doi: 10.1038/s41598-026-52148-9 (PMC13172392; doi:10.1038/s41598-026-52148-9)
Supplement: Supplementary file 2 — Supplementary Material 2 [file 41598_2026_52148_MOESM2_ESM.docx]

# Supplementary materials

## Thresholding algorithm

The thresholding algorithm used applies the following scipy and scikit-image Python functions to WSIs downsampled to a resolution of 8.0 μm per pixel:

1. convolution (scipy.signal.convolve2d) using the isotropic “Mehrstellen” nine-point stencil of the two-dimensional Laplacian operator^1^ as a kernel;

2. Otsu’s thresholding (skimage.filters.threshold_otsu) on the convoluted image to find the optimal threshold, creating a binary image using values of the convoluted image greater than the threshold,

3. binary closing (skimage.morpholoy.binary_closing, structure=skimage.morphology.disk(9)) on the resulting binary image;

4. binary opening (skimage.morphology.binary_opening, structure=skimage.morphology.disk(5)),

5. filling any small holes (scipy.ndimage.morphology.binary_fill_holes);

6. removing small objects (skimage.morphology.remove_small_objects, min_size=300, connectivity=4);

7. removing thin objects (remove any region with skimage.measure.regionprops.minor_axis_length > 300);

8. removing hues, saturations, and values outside a specified range (skimage.color.rgb2hsv, generating a binary structure with structure=scipy.ndimage.morphology.generate_binary_structure(rank=2, connectivity=2), labeling regions with with scipy.ndimage.label(structure=structure)), then keeping only regions with median h between 0 and 0.1 or between 0.7 and 1.0, median s greater than 0.08, and median v greater than 0.6);

9. clearing borders (skimage.segmentation.clear_border, buffer_size=0, bgval=0).

##

## Augmentations

Three groups of augmentations from the Albumentations Python library (version 1.3.1) were used. Each group had a 50% probability to be applied, and within the groups the augmentations were applied with probability p specified below:

**Basic augmentations (p=0.5):**

- Vertical flip: albumentations.VerticalFlip (probability p=0.5)
- Horizontal flip: albumentations.HorizontalFlip (p=0.5)
- Random 90° rotations: albumentations.RandomRotate90 (p=1.0)

**Advanced augmentations (p=0.5):**

- Unsharp masking: albumentations.UnsharpMask (p=0.5)
  - blur_limit: [1, 51], alpha: [0.5, 1.0]
- Gaussian blurring: albumentations.GaussianBlur (p=0.5)
  - blur_limit: [1, 9]
- Color jitter: albumentations.ColorJitter (p=0.5)
  - brightness: [0.8, 1.2], contrast: [0.5, 1.5], saturation: [0.5, 1.5], hue: [-0.05, 0.05]
- Gamma correction: albumentations.RandomGamma (p=0.5)
  - gamma_limit: [80, 120]
- Random tone curve adjustment: albumentations.RandomToneCurve (p=0.5)
  - scale: 0.2

**Noise augmentations (p=0.5):**

- Gaussian noise: albumentations.GaussNoise (p=0.5)
  - var_limit: [1, 50]
- Multiplicative noise: albumentations.MultiplicativeNoise (p=0.5)
  - multiplier: [0.95, 1.05], element-wise
- Camera sensor noise: albumentations.ISONoise (p=0.5)
  - color_shift: [0.01, 0.05], intensity: [0.1, 0.5]
- Image compression artifacts: albumentations.ImageCompression (p=0.5)
  - quality_lower: 70

# References

1. Patra, M. & Karttunen, M. Stencils with isotropic discretization error for differential operators. *Numer. Methods Partial Differ. Equ.* **22**, 936–953 (2006).
