# Supplementary material for: The impact of tissue detection on diagnostic artificial intelligence algorithms in prostate digital pathology
Source: Sci Rep. 2026 May 13;16:14968. doi: 10.1038/s41598-026-52148-9 (PMC13172392; doi:10.1038/s41598-026-52148-9)
Supplement: Supplementary file 3 — Supplementary Material 3 [file 41598_2026_52148_MOESM3_ESM.docx]

**Supplementary Figure 1:** The concordance between the predictions of ISUP grade by two versions of the Gleason grading model and the reference grading by pathologists, measured by Cohen’s quadratically weighted kappa statistic. Results are shown based on tissue detection on the validation slides using AI-based and thresholding-based methods. One of the Gleason grading models was trained using tissue segmentation masks that had been generated using thresholding (“thresholding model”) and one was trained using tissue segmentation masks that had been generated using the tissue detection AI (“AI model”), and each was evaluated using segmentation masks generated with each of the methods (“thresholding tiles”, “AI tiles”). The dots indicate point estimates on the entire dataset and the whiskers indicate 95% CIs. Confidence intervals were estimated with bootstrapping using 1000 replicates. Only cases where both models were able to detect any tissue were included, see **Table 2**. Synlab Finland and Synlab Switzerland had reference grading per anatomical location and SPROB had reference grading per patient; WSIs from these cohorts were pooled to get predictions at location and patient levels, respectively.

**Supplementary Figure 2:** The concordance between the predictions of ISUP grade by two versions of an older Gleason grading model than what was used in other analyses and the reference grading by pathologists, measured by Cohen’s quadratically weighted kappa statistic. Results are shown based on tissue detection on the validation slides using AI-based and thresholding-based methods. The model’s ability to distinguish between benign and malignant slides differed heavily across the cohorts, with Aarhus, Synlab France and Stockholm3 heavily favouring sensitivity; Radboud strongly favouring specificity; and the rest being more balanced. Moreover, the ability to accurately predict Gleason grade (and by extension, ISUP grade) was generally poor, with a tendency to predict too high for most but not all cohorts. Despite the heterogeneity, the effect of tissue detection on the analyzed slides remained insignificant for all cohorts. The dots indicate point estimates on the entire dataset and the whiskers indicate 95% CIs. Confidence intervals were estimated with bootstrapping using 1000 replicates.
